# Ginger and Turmeric Essential Oils for Weed Control and Food Crop Protection

**DOI:** 10.3390/plants8030059

**Published:** 2019-03-10

**Authors:** María Dolores Ibáñez, María Amparo Blázquez

**Affiliations:** Departament de Farmacologia, Facultat de Farmàcia, Universitat de València, Avd. Vicent Andrés Estellés s/n, 46100 Burjassot, Valencia, Spain; mijai@alumni.uv.es

**Keywords:** ginger, turmeric, essential oils, gas chromatography–mass spectrometry, weed control, food crops, phytotoxicity

## Abstract

Ginger and turmeric are two food ingredients that are in high demand due to their flavor and positive effects on health. The biological properties of these spices are closely related to the aromatic compounds they contain. The chemical compositions of their essential oils and their *in vitro* phytotoxic activity against weeds (*Portulaca oleracea*, *Lolium multiflorum*, *Echinochloa crus-galli*, *Cortaderia selloana*, and *Nicotiana glauca*) and food crops (tomato, cucumber, and rice) were studied. Forty-one compounds, accounting for a relative peak area of 87.7% and 94.6% of turmeric and ginger essential oils, respectively, were identified by Gas Chromatography–Mass Spectrometry analysis. Ginger essential oil with α-zingiberene (24.9 ± 0.8%), β-sesquiphelladrene (11.7 ± 0.3%), ar-curcumene (10.7 ± 0.2%), and β-bisabolene (10.5 ± 0.3%) as the main compounds significantly inhibited the seed germination of *P. oleracea*, *L*. *multiflorum*, and *C. selloana* at the highest dose (1 µL/mL) assayed, as well as the hypocotyl and radicle growth of the weeds. Turmeric essential oil with ar-turmerone (38.7 ± 0.8%), β-turmerone (18.6 ± 0.6%), and α-turmerone (14.2 ± 0.9%) as principal components significantly inhibited the seed germination of *C. selloana* and hypocotyl and radicle growth of weeds (the latter in particular) at the highest dose, whereas it did not affect either the seed germination or seedling growth of the food crops. Turmeric essential oil can be an effective post-emergent bioherbicide against the tested weeds without phytotoxicity to crops.

## 1. Introduction

Human consumption of herbs and spices began in 5000 BC [1] and has continued until today due to the fact that these products are added to a great variety of food, especially ready-to-eat foods [2]. The world production of spices increased from 424.3 tons in 1961 to 2,413,284 tons in 2016 [3].

Herbs and spices offer a wide range of flavors that increase sensory variety in food and beverages without additional energy [4] while providing health benefits, due mainly to their antioxidant properties [5]. Several spices are dietary agents with anticancer properties due to containing compounds like curcumin, gingerol, anethole, or zerumbone, which are powerful inhibitors of nuclear factor κB (NF-κB), protein complex involved in DNA transcription [6].

Ginger (*Zingiber officinale* Rosc.) and turmeric (*Curcuma longa* L.), two powerful spices, have been widely used for both culinary and medical purposes. Ginger is an underground stem (rhizome) of a perennial herb and is used as a spice for pickles, candies, and as a preserve [7], while turmeric, popularly called “Indian saffron” [8], is also a dried rhizome of a herbaceous plant that imparts a distinctive flavor and orange color to food. 

Ginger is able to exhibit antioxidant properties comparable to those of the standard synthetic antioxidants butylated hydroxyanisole (BHA) and butylated hydroxytoluene (BHT), indicating that it may reduce or delay the progression of diseases related with oxidative stress [9]. Ginger constituents can relieve arthritic pain by interfering in the inflammatory cascade and the vanilloid nociceptor [10]. Furthermore, *in vitro*, *in vivo*, and epidemiological studies have corroborated that ginger and its active compounds are effective against a wide variety of human cancers, like gastric, pancreatic, liver, and colorectal cancer, as well as cholangiocarcinoma [11]. These facts, together with its antidiabetic [12], lipid-lowering, anti-obesity, and cardioprotective effects [10], make ginger an excellent nutraceutical among spices. 

Beneficial health effects of turmeric and especially of curcumin—an orange-yellow-colored, lipophilic polyphenol substance—have been reported [13]. Curcumin is able to effectively modulate molecular targets that have a role in many phases of cancer development [13,14]. It also has a beneficial effect on inflammation, diabetes, and neurodegenerative diseases [15]. In relation to this, it has been observed that curcumin alleviates airway inflammation and ameliorates the expression of pro-inflammatory cytokines through the phosphorilation of nuclear factor-erythroid 2 related factor 2 together with the expression of heme oxygenase-1 (Nrf2/HO-1 signaling pathway) [16]; curcumin, being an amyloid-binding probe, reduces chronic inflammation, facilitates resolution of inflammation, and reduces lipid peroxidation that is correlated with synapse loss, causing it to have beneficial effects in Alzheimer’s disease [17].

Essential oils of these spices also have interesting pharmacological activities, for instance, both essential oils are *in vivo* antimutagenic and anticarcinogenic substances. Ginger essential oil is able to significantly increase the levels of phase II carcinogen-metabolizing enzymes uridine 5′-diphospho-glucuronyl transferase and glutathione-S-transferase [18], and turmeric essential oil inhibits enzymes (p450) such as the cytochromes CYP1A1, CYP1A2, CYP2B, CYP2A, CYP2D, and CYP3A involved in the activation of carcinogens [18]. Furthermore, ginger essential oil might be an effective dietary supplement to ameliorate non-alcoholic fatty liver disease and related metabolic diseases throughout the regulation of hepatic lipid synthesis, antioxidant enzymes, and inflammatory factors, which involves modulation of the hepatic sterol regulatory element binding the protein SREBP-1c and CYP2E1-mediated pathway [19].

Further investigation is necessary in order to know about other potential activities of these essential oils, not only in medicine but also in other remarkable areas like harvest and post-harvest protection of food and crops. Regarding this, turmeric essential oil in edible coatings has been found to improve the shelf-life of cherry tomatoes and raw poultry milk [20,21]. It has shown toxic and fumigant activity against stored grain insects *Sitophilus oryzae* L. and *Rhyzopertha dominica* F. [22] and antifungal and antimycotoxigenic activities against *Fusarium verticillioides* and *F. graminearum*, as well as fumonisins (B1 and B2) and zearalenone production [23,24]. Ginger essential oil was also found to be effective against fungi such as *Aspergillus flavus*, completely inhibiting conidial germination at 10 µg/mL of ginger essential oil as well as aflatoxin production at 15 µg/mL [25]. Finally, 0.3% (v/v) ginger essential oil exhibited complete inhibition against the phytopathogenic fungi *Alternaria panax*, *Botrytis cinerea*, *Cylindrocarpon destructans*, *F. oxysporum*, *Sclerotinia sclerotiorum*, and *S. nivalis* responsible for ginseng root rot disease [26].

These studies corroborated the insecticidal and antifungal properties of ginger and turmeric essential oils and their beneficial effects on food crops. However, weeds are also responsible for lost production of food crops. Regarding this, weed management in ginger as well as the herbicidal activity against *Parthenium hysterophorus* of both hexane and aqueous extracts from ginger has been studied [27,28]. The phytotoxic effects of *Curcuma* spp., like *C. zedoaria* essential oil with 1,8-cineole (15.8%) and *epi*-curzerenone (18.2%) as the main compounds, has also been demonstrated against both lettuce and tomato [29]; *C. longa* extracts with curcuminoids are able to inhibit the germination and growth of *Bidens pilosa* [30]. Therefore, the aims of this study were as follows: firstly, to determine through Gas Chromatography–Mass Spectrometry analysis the chemical composition of commercial ginger and turmeric essential oils in order to know their main constituents; secondly, to observe their *in vitro* herbicidal effects against the seed germination and seedling growth of common ragweed (*Portulaca oleracea* L.), Italian ryegrass (*Lolium multiflorum* Lam.), barnyardgrass (*Echinochloa crus-galli* (L.) Beauv.), pampas grass (*Cortaderia selloana* (Schult. & Schult. f.) Asch. & Graebn.), and tree tobacco (*Nicotiana glauca* Graham); and finally, to determine whether these essential oils have phytotoxic effects on food crops like tomato (*Solanum lycopersicum* L.), cucumber (*Cucumis sativus* L.), and rice (*Oryza sativa* L.). 

## 2. Results and Discussion

### 2.1. Chemical Composition of Ginger and Turmeric Essential Oils 

Forty-one compounds in commercial ginger and turmeric essential oils accounting for 94.60% and 87.67% of the total composition, respectively, were identified by Gas Chromatography–Mass Spectrometry analysis. The components were clustered (Table 1) as homologous series of monoterpene hydrocarbons, oxygenated monoterpenes, sesquiterpene hydrocarbons, oxygenated sesquiterpenes, and others and listed according to Kovat’s retention index calculated in GC on an apolar HP-5MS column. 

Sesquiterpene compounds represented the main phytochemical group found in both ginger and turmeric essential oils, of which sesquiterpene hydrocarbons (59.6 ± 0.3%) with seven compounds identified were the major set in ginger essential oil, while oxygenated sesquiterpenes (73.9 ± 1.4%) were the principal ones in turmeric essential oil with six components recognized (Table 1). It is well known that hydrocarbons and oxygenated sesquiterpenes not only have a higher structural diversity than monoterpene, but also contribute to a noteworthy extent to the special aroma and flavor of essential oils. [31]

The sesquiterpene hydrocarbons α-zingiberene (24.9 ± 0.8%), β-sesquiphelladrene (11.9 ± 0.3%), ar-curcumene (10.7 ± 0.2%) and β-bisabolene (10.5 ± 0.3%), detected in lower percentages in turmeric essential oil (2.6 ± 0.1, 2.2 ± 0.0, 1.4 ± 0.1, and 0.6 ± 0.0%, respectively), were the main compounds in ginger essential oil. The results obtained were similar to those of recent research [32] in which zingiberene (16.3%), curcumene (12.4%), sesquiphellandrene (11.4%), and β-bisabolene (4.2%) were also found to be the major components of ginger essential oil from Ankara (Turkey) or with samples from Ecuador, in which α-zingiberene (17.4%) and β-sesquiphelladrene (6.7%) were between the main sesquiterpene hydrocarbons [33].

Although zingiberene was the major compound in essential oils coming from both fresh and dried ginger rhizomes from Trivandrum (India), fresh ginger essential oil contained more oxygenated sesquiterpenes compared to the dried one which contained large amounts of the sesquiterpene hydrocarbons ar-curcumene (11.0%), β-bisabolene (7.2%), sesquiphellandrene (6.6%), and δ-cadinene (3.5%) [34].

Zingiberene, the chief component of the *Z. officinale* essential oil here analyzed, is a monocyclic sesquiterpene hydrocarbon with natural antioxidant and cytotoxic activities: it is capable of protecting against H_2_O_2_-induced cytotoxicity and oxidative DNA damage in neuronal cells [35] as well as inhibiting the growth of lymphocytic cells in a dose-dependent manner [36]. Furthermore, high zingiberene content in tomato plants provides resistance against arthropod pests including spider mite (*Tetranychus urticae*) and whitefly (*Bemisia tabaci*) [37,38]. On the other hand, β-sesquiphelladrene, the main isomer of zingiberene and second main compound in the ginger essential oil here analyzed, has antiviral and antifertility effects [38] as well as anticancer potential by inducing apoptosis through mitochondrial pathways [39].

However, different freezing rates and thawing methods can significantly affect the composition of ginger essential oil: gingerol (3.6%) and zingerone (18.3%), the main spicy compounds of fresh ginger, reached maximum percentages when ginger was thawed by an infrared method (gingerol, 7.3%) or after thawing ginger using an infrared–microwave (zingerone, 38.3%) method [40]. These results indicated that the essential oil here analyzed and employed in phytotoxic assays was not obtained from ginger rhizome by infrared or infrared–microwave methods.

On the other hand, ar-turmerone (38.7 ± 0.8%), β-turmerone (18.6 ± 0.6%), and α-turmerone (14.20 ± 0.86%), which were not found in ginger oil, were the leading components of turmeric essential oil. The rest of the sesquiterpenes did not reach 1% in either essential oil analyzed (Table 1). These results coincide with those of previous studies in which ar-turmerone, α-turmerone, and β-turmerone were also found to be the leading compounds in turmeric essential oil [41]. However, similarly to ginger essential oil, other studies have reported changes in the chemical composition of turmeric essential oil depending on the biological raw material (fresh or dried) employed, with ar-turmerone (24.4%), α-turmerone (20.5%) and β-turmerone (11.1%), or ar-turmerone (49.1 ± 3.5%) and β-turmerone (16.8 ± 0.4%) [42] in fresh *C. longa* rhizome and ar-turmerone (21.4%) and the sesquiterpene hydrocarbons α-santalene (7.2%) and ar-curcumene (6.6%) in turmeric essential oil obtained from dry rhizome [42]. Higher percentages of the sesquiterpene hydrocarbons ar-curcumene (7.8%), zingiberene (4.2%), and β-sesquiphelladrene (22.8%) were found in turmeric essential oil obtained by hydrodistillation from *C. longa* leaves [43], confirming the GC–MS analysis [44] that our essential oil was obtained from fresh rhizomes by hydrodistillation.

The therapeutic potential of ar-turmerone has been extensively studied due to its numerous beneficial effects such as anti-inflammatory and cytotoxic effects in the treatment of various neurodegenerative disorders [45,46]. Regarding pest control, ar-turmerone has also been observed to protect against insect and mite infestation; consequently, it has been incorporated into packaging material in order to avoid pest penetration of packaged products [47]. Specially, ar-turmerone has been observed to be highly toxic against maize weevil (*Sitophilus zeamais*) and fall armyworm (*Spodoptera frugiperda*) at low doses [48].

Monoterpene hydrocarbons were the following main phytochemical group with eight (19.8 ± 0.1%) and nine (5.4 ± 0.7%) compounds identified in ginger and turmeric essential oils, respectively (Table 1). Camphene (11.6 ± 0.3%), followed by limonene (3.2 ± 0.1%), α-pinene (2.7 ± 0.0%), and myrcene (1.3 ± 0.0%), was the main compound in ginger essential oil, while α-phellandrene (4.3 ± 0.4%) was the principal component in turmeric essential oil (Table 1).

1,8-Cineole (1.0 ± 0.0%) was the only oxygenated monoterpene detected in turmeric essential oil. In contrast, this fraction, with ten oxygenated monoterpenes identified, was qualitatively the main phytochemical group found in ginger essential oil. 1,8-Cineole (3.0 ± 0.1%), followed by geranial (3.2 ± 0.0%) and neral (2.1 ± 0.1%), were the main compounds (Table 1).

Recent studies [49] showed that essential oils containing 1,8-cineole are toxic against the tick species *Rhipicephalus* (Boophilus) *microplus*, and neral and geranial have exhibited anti-inflammatory activity through significant and similar inhibition of the gene NLRP-3 inflammasome-mediated IL-1β secretion, showing use as functional food ingredients [50].

Finally, other compounds such as 6-methyl-5-hepten-2-one (2.1 ± 0.1%), 2-nonanone (0.1 ± 0.0%), and 2-undecanone (0.2 ± 0.0%) were only identified in ginger essential oil (Table 1).

### 2.2. Seed Germination and Seedling Growth Inhibition of P. oleracea, L. multiflorum, E. crus-galli, C. selloana, and N. glauca with Ginger and Turmeric Essential Oils

**Figure 1:**
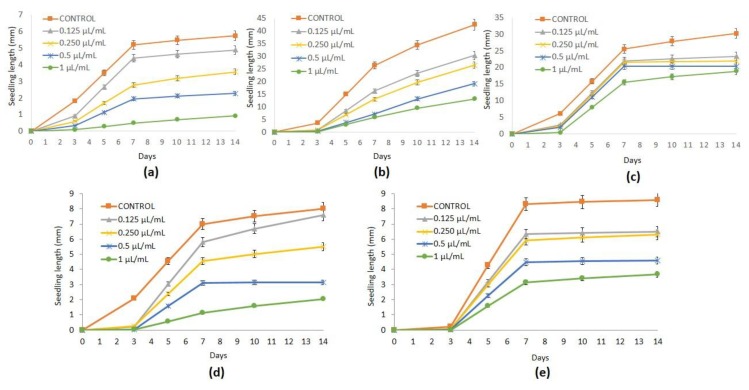
Values of seedling lenght (mm) (mean ± SE) of *Portulaca oleracea* (**a**), *Lolium multiflroum* (**b**), *Echinochloa crus-galli* (**c**), *Cortaderia selloana* (**d**) and *Nicotiana glauca* (**e**) control and treated with ginger essential oil at 0.125, 0.25, 0.5 and 1 µL/mL.

**Figure 2:**
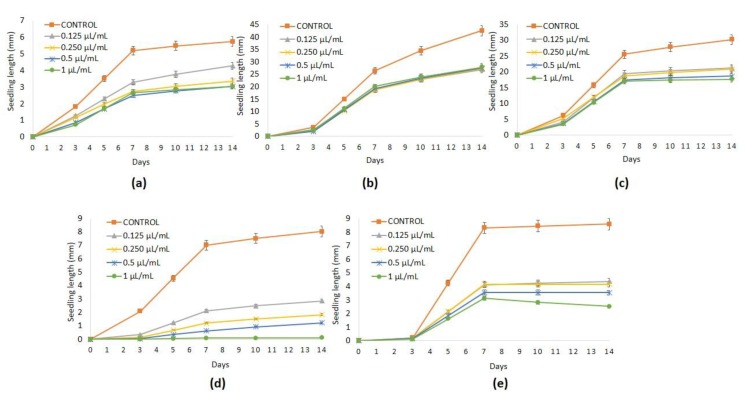
Values of seedling lenght (mm) (mean ± SE) of *Portulaca oleracea* (**a**), *Lolium multiflroum* (**b**), *Echinochloa crus-galli* (**c**), *Cortaderia selloana* (**d**) and *Nicotiana glauca* (**e**) control and treated with turmeric essential oil at 0.125, 0.25, 0.5 and 1 µL/mL.

As several studies have indicated that essential oils may be promising herbicides [51], the effects of ginger and turmeric essential oils were tested (Table 2 and Table 3 and Figure 1 and Figure 2) against the seed germination and seedling growth of *P. oleracea*, *L. multiflorum*, *E. crus-galli*, *C. selloana*, and *N. glauca*.

Turmeric essential oil had no phytotoxic effects on the seed germination of *P. oleracea*, *L. multiflorum*, *E. crus-galli*, and *N. glauca* at all doses (0.125, 0.25, 0.50, and 1 µL/mL) assayed; however, significant inhibition of the seed germination of *C. selloana* was achieved in a dose-dependent manner, reaching 81.71% of reduction at the highest dose (1 µL/mL) tested (Table 2).

Previous studies showed that *P. oleracea*, *L. multiflorum*, and *E. crus-galli* were sensitive to winter savory (*Satureja montana* L.), which exerted a total inhibitory effect on the seed germination of the three weeds at all doses (0.125, 0.25, 0.50, and 1 µL/mL) tested, and peppermint (*Mentha piperita* L.), which completely inhibited the seed germination of *L. multiflorum* and significantly affected the seed germination of *P. oleracea* and *E. crus-galli* at the highest dose (1 µL/mL) applied [52].

Regarding ginger essential oil, although there was no significant inhibitory effect on the seed germination of *E. crus-galli* and *N. glauca*, a remarkable decrease in the seed germination of *P. oleracea*, *L. multiflorum*, and *C. selloana* was observed at the highest dose—reductions of 45.35%, 46.67%, and 43.91%, respectively— in relation to the control (Table 2).

In the seedling evolution, ginger essential oil caused a significant dose-dependent inhibition of the hypocotyl development of *P. oleracea*, *L. multiflorum*, *C. selloana*, and *N. glauca*, reaching high reduction percentages of 82.74%, 66.85%, 73.68%, and 63.77%, respectively, at the highest dose (1 µL/mL) in comparison to the control (Table 3). However, no significant reduction in *E. crus-galli* hypocotyl growth was observed at any dose assayed (0.125, 0.25, 0.50, and 1 µL/mL) with respect to the control (Table 3, Figure 1C).

Ginger essential oil also considerably influenced the radicle progress of the five selected weeds. The radicle development of *P. oleracea* was significantly reduced by 57.22% and 86.06% relative to the control after the application of ginger essential oil at 0.5 and 1 µL/mL, respectively; this was similar to *L. multiflorum*, whose radicle enlargement was decreased at these doses between 60.23% and 72.36% (Table 3, Figure 1A,B). The radicle elongation of *E. crus-galli* significantly declined at these doses between 39.95% and 50.61% (Table 3, Figure 1C). A noteworthy reduction in radicle development was achieved in *C. selloana*, which experienced a decline percentage of 75.26% at the highest dose (1 µL/mL) assayed (Table 3, Figure 1D); finally, a significant inhibition of 48.32% of the radicle growth of *N. glauca* was observed at the highest dose (1 µL/mL) applied (Table 3, Figure 1E).

Furthermore, other *Zingiber* spp. have also shown phytotoxicity against different weeds; for instance, *Z. zerumbet* Smith, with zerumbone (74.82%) as its major compound, affected the seedling growth of *Philaris minor* Retz. in a concentration-dependent manner, achieving inhibition of both the hypocotyl and radicle development at 1000 ppm and showing less or no effect on the germination of seeds of *Triticum aestivum* L. [53].

Turmeric essential oil, with the exception of the radicle elongation of *P. oleracea*, significantly inhibited both hypocotyl and radicle growth of the selected weeds at all doses (0.125, 0.25, 0.50, and 1 µL/mL) assayed. The hypocotyl development was reduced without significant differences between doses applied to reach percentages of 56.55% (*P. oleracea*), 40.45% (*L. multiflorum*), 39.33% (*E. crus-galli*), 97.83% (*C. selloana*), and 86.23% (*N. glauca*) (Table 3). The radicle elongation of *L. multiflorum* and *E. crus-galli* was significantly reduced at all doses of turmeric essential oil, reaching 36.74% and 44.41%, respectively, at the highest dose tested. *C. selloana* was again the most sensitive species to turmeric essential oil with percentages of radicle growth inhibition of 77.32%, 81.44%, 86.08%, and 99.74% at the doses of 0.125, 0.25, 0.50, and 1 µL/mL, whereas *N. glauca* reached a percentage of 51.42% at the highest dose applied.

Ginger and turmeric essential oils are not suitable as a potent pre-emergent treatment in the control of *P. oleracea*, *E. crus-galli*, and *L. multiflorum* because other essential oils such as oregano essential oil with carvacrol (60.4 ± 0.1%), *p*-cymene (15.5 ± 0.0%), and γ-terpinene (5.2 ± 0.0%) or winter savory essential oil with carvacrol (43.3 ± 0.1%) and thymol (23.2 ± 0.1) as main compounds can completely inhibit the germination of these three weeds at all doses (0.125–1 µL/mL) applied [52,54]. These essential oils have similar herbicidal potential to *Thymus mastichina* essential oil with 1,8-cineole (49.5 ± 0.4%), linalool (5.7 ± 0.0%), and α-terpineol (5.6 ± 0.0%), which showed significant effects in seedling length depending on the weed and dose [54]. In addition, turmeric essential oil could be used as a bioherbicide in the control of the invasive species *C. selloana.* Their use as promising post-emergent alternatives will depend on the phytotoxicity of these essential oils in food crops.

### 2.3. Seed Germination and Seedling Growth Effect of Ginger and Turmeric Essential Oils in Tomato, Cucumber, and Rice

Seed germination of tomato, cucumber, and rice was not affected at any dose (0.125, 0.25, 0.50, and 1 µL/mL) applied of ginger essential oil (Table 4).

**Figure 3:**
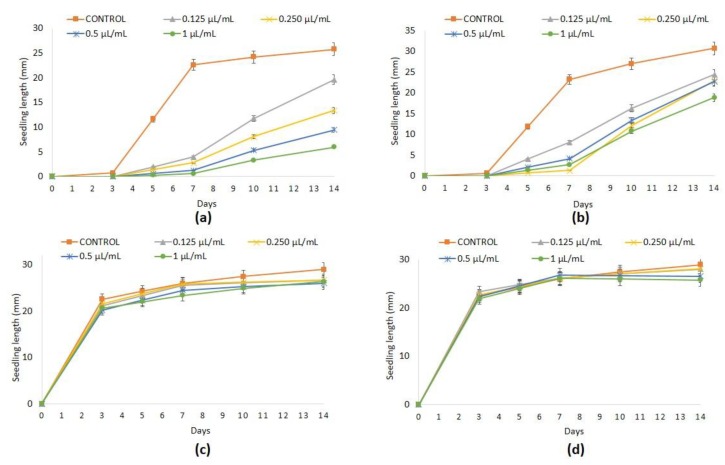
Values of seedling lenght (mm) (mean ± SE) of tomato control and treated with ginger (**a**) and turmeric (**b**) essential oils and cucumber control and treated with ginger (**c**) and turmeric (**d**) essential oils at 0.125, 0.25, 0.5 and 1 µL/mL.

**Figure 4:**
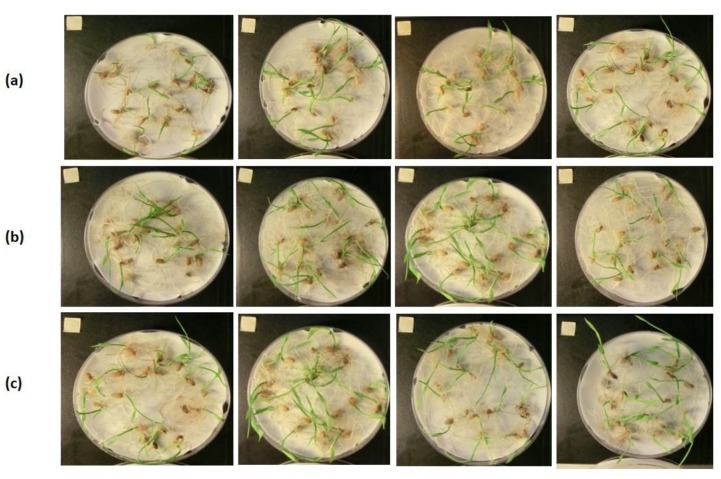
Rice control (**a**) and treated with ginger (**b**) and turmeric (**c**) essential oils at 0.125, 0.25, 0.5 and 1 µL/mL in day 14 of treatment.

Phytotoxic effects observed at 1 µL/mL of ginger essential oil in *P. oleracea* (45.35%) and *L. multiflorum* (46.67%) (Table 2)—weeds commonly affecting tomato crops [55]—were not reproduced in tomato germination, but, unfortunately, both hypocotyl and radicle development were significantly inhibited (Table 4, Figure 3A). These results agree with those of previous work in which seed germination of soybean was not inhibited by the aqueous extract of ginger rhizome at the doses assayed, whereas the hypocotyl and radicle length were reduced at the higher doses applied [56]. On the other hand, neither seed germination nor the hypocotyl growth of cucumber and rice were affected by ginger essential oil at any dose (0.125, 0.25, 0.50, and 1 µL/mL) assayed. The radicle elongation of cucumber was decreased in a dose-dependent manner up to a percentage of 21.44% at the highest dose (Table 4, Figure 3C). Slight differences in the radicle lengths of rice among the measurements were observed, but the data are not presented due to the difficulty of accurately measuring curved radicles (Figure 4).

Promising results were obtained with turmeric essential oil against the food crops tested. Neither seed germination nor the hypocotyl growth of tomato, cucumber, and rice were significantly affected by the application of turmeric essential oil at any dose (0.125, 0.25, 0.50, and 1 µL/mL) (Table 4, Figure 3B,D). The radicle elongation of tomato, cucumber (Table 4), and rice (Figure 4) was also not affected by turmeric essential oil. Previous studies have also reported the harmlessness of turmeric essential oil *versus* other food crops: for instance, chickpea, in which no adverse effect was observed in either seed germination or seedling growth [57]. However, other *Curcuma* spp. like *C. zedoaria* have been shown to inhibit the seed germination of lettuce and tomato in a dose-dependent manner (0.00%, 0.25%, 0.50%, 0.75%, and 1.00%) as well as to delay their growth, damaging the root in particular. These results are due to the different chemical compositions of these essential oils, with ar-turmerone (38.7 ± 0.8%), β-turmerone (18.6 ± 0.6%), and α-turmerone (14.2 ± 0.9%) being the main compounds in the turmeric essential oil here analyzed (Table 1), and 1,8-cineole (15.8%) and *epi*-curzerenone (18.2%) being those in *C. zedoaria* essential oil [29].

It is interesting to note that at the highest dose assayed, turmeric essential oil was able to significantly reduce the hypocotyl development of *P. oleracea* (Table 3) as well as both the hypocotyl and radicle growth of *L. multiflorum*, *E. crus-galli*, *C. selloana*, and *N. glauca*, without the phytotoxic effects shown by other essential oils such as rosemary (*Rosmarinus officinalis* L.), winter savory (*Satureja hortensis* L.), and bay (*Laurus nobilis* L.) in tomato [58].

## 3. Materials and Methods

### 3.1. Essential Oils

Commercial samples of ginger (*Zingiber officinale* Rosc.) (Batch 0F26093; Exp. date 04/2022; 1016 Indonesia) and turmeric (*Curcuma longa* L.) (Batch 0F27683; Exp. date 10/2021; 0516 India) essential oils obtained from rhizome and root, respectively, were supplied by Pranarôm S.A. Both were stored at 4 ᵒC until chemical analysis and phytotoxic assays were carried out.

### 3.2. Weed and Food Crop Seeds

Mature seeds of the weeds common ragweed (*Portulaca oleracea* L.), Italian ryegrass (*Lolium multiflorum* Lam.), and barnyardgrass (*Echinochloa crus-galli* (L.) Beauv.) were purchased from Herbiseed (website: www.herbiseed.com), and those of pampas grass (*Cortaderia selloana* (Schult. & Schult. f.) Asch. & Graebn.) and tree tobacco (*Nicotiana glauca* Graham) were supplied by the Botanical Garden of Valencia.

Mature seeds of the food crops “Muchamiel” tomato (*Solanum lycopersicum* L.) and cucumber (*Cucumis sativus* L.) were obtained from Intersemillas S.A. “Albufera-type” rice (*Oryza sativa* L.) seeds were acquired from Copsemar in Sueca (Valencia, Spain).

### 3.3. Gas Chromatography–Mass Spectrometry Analysis

GC–MS analysis was carried out using a 5977A Agilent mass spectrometer and a gas chromatograph (Agilent 7890B) apparatus equipped with an Agilent HP-5MSi (30 m long and 0.25 mm i.d. with 0.25 µm film thickness) capillary column (95% dimethylpolysiloxane/5% diphenyl). The column temperature program was 60 ᵒC for a duration of 5 min, with 3 ᵒC/min increases to 180 ᵒC, then 20 ᵒC/min increases to 280 ᵒC, which was maintained for 10 min. The carrier gas was helium at a flow rate of 1 mL/min. Split mode injection (ratio 1:30) was employed. Mass spectra were taken over the m/z range 30–650 with an ionizing voltage of 70 eV. The resulting individual compounds were identified by MS and their identity was confirmed by comparison of their Kovat’s retention index calculated using co-chromatographed standard hydrocarbons relative to C_8_–C_32_ *n*-alkanes and mass spectra with reference samples or with data already available in the NIST 11 mass spectral library and in the literature [59].

### 3.4. In Vitro Assays: P. oleracea, L. multiflorum, E. crus-galli, C. selloana, N. glauca, Tomato, and Rice Seed Germination and Seedling Growth with Essential Oils

Sets of 20 seeds each with five replicates per treatment were homogenously distributed in Petri dishes (9 cm diameter) between two layers of filter paper (Whatman No.1) moistened with 4 mL of distilled water and with 0 (control), 0.125, 0.250, 0.5, and 1 µL/mL of ginger and turmeric essential oils. Petri dishes were sealed with parafilm and incubated in an Equitec EGCS 301 3SHR model germination chamber, according to previous assays [60], alternating 30.0 ± 0.1ºC 16 h in light and 20.0 ± 0.1ºC 8 h in dark and with (*E. crus-galli*, *C. selloana*, *N. glauca,* cucumber, and rice) and without (*P. oleracea*, *L. multiflorum*, tomato) humidity. To evaluate the herbicidal activity of the essential oils, the number of germinated seeds was counted and compared with that of untreated seedlings. Emergence of the radicle (≥ 1 mm) was used as an index of germination and seedling length (hypocotyl and/or radicle) data were recorded after 3, 5, 7, 10, and 14 days in each replicate.

### 3.5. Statistics

Experiments were performed with five replicates. Data were subjected to one-way analysis of variance (ANOVA) using SPSS statistics 22 software. Tukey’s post hoc test was used when variances remained homogeneous (Levene’s test) and T3 Dunnett’s post hoc test was employed if not, assuming equal variances. Differences were considered to be significant at *p* ≤ 0.05.

## 4. Conclusions

Essential oils from ginger and turmeric, two health-promoting spices, could be used in weed control. Ginger essential oil with high contents of the sesquiterpene hydrocarbons α-zingiberene (24.9 ± 0.8%), β-sesquiphelladrene (11.9 ± 0.3%), ar-curcumene (10.7 ± 0.2%), and β-bisabolene (10.5 ± 0.3%) may be used as a pre-emergent bioherbicide in the control of *P. oleracea* and *L. multiflorum* in tomato, cucumber, and rice crops, whereas turmeric essential oil with the oxygenated sesquiterpenes ar-turmerone (38.7 ± 0.8%), β-turmerone (18.6 ± 0.6%), and α-turmerone (14.2 ± 0.9%) can be applied as a post-emergent substance against the weeds tested since no significant phytotoxic effects in tomato, cucumber, or rice were observed. Turmeric essential oil could be a promising alternative in the management of the invasive species *C. selloana*. More weeds and higher doses of turmeric essential oil must be tested in order to determine any selective herbicide effect.

## Figures and Tables

**Table 1:** Chemical composition of commercial ginger and turmeric essential oils.

RI_Cal_	RI_Ref_	Compound	Ginger Essential Oil Relative Area (%)	Turmeric Essential Oil Relative Area (%)	Identification
Monoterpene hydrocarbons			19.8 ± 0.5	5.4 ± 0.7	
919	926	Tricyclene	0.2 ± 0.0	-	RI, MS
932	939	α-Pinene	2.7 ± 0.0	0.2 ± 0.0	RI, MS
948	954	Camphene	11.6 ± 0.3	-	RI, MS
973	979	β-Pinene	0.2 ± 0.0	-	RI, MS
987	990	Myrcene	1.3 ± 0.04	0.1 ± 0.0	RI, MS
998	1002	α-Phellandrene	0.2 ± 0.0	4.3 ± 0.4	RI, MS
1004	1011	δ-3-Carene	-	0.1 ± 0.0	RI, MS
1013	1017	α-Terpinene	-	0.1 ± 0.0	RI, MS
1021	1024	*p*-Cymene	-	0.5 ± 0.1	RI, MS
1026	1029	Limonene	3.2 ± 0.1	0.2 ± 0.0	RI, MS
1056	1059	γ-Terpinene	-	0.2 ± 0.0	RI, MS
1083	1088	Terpinolene	0.3 ± 0.0	0.2 ± 0.0	RI, MS
Oxygenated monoterpenes			11.8 ± 0.2	1.0 ± 0.0	
1029	1031	1,8-Cineole	3.0 ± 0.1	1.0 ± 0.0	RI, MS
1095	1094	Linalool	0.8 ± 0.0	-	RI, MS
1137	1146	Camphor	0.2 ± 0.0	-	RI, MS
1149	1153	Citronellal	0.2 ± 0.0	-	RI, MS
1171	1177	Terpinen-4-ol	0.2 ± 0.0	-	RI, MS
1188	1188	α-Terpineol	0.7 ± 0.1	-	RI, MS
1236	1238	Neral	2.1 ± 0.1	-	RI, MS
1267	1267	Geranial	3.2 ± 0.0	-	RI, MS
1279	1288	Bornyl Acetate	0.9 ± 0.0	-	RI, MS
1378	1381	Geranyl Acetate	0.6 ± 0.0	-	RI, MS
Sesquiterpene hydrocarbons			59.6 ± 0.1	7.2 ± 0.0	
1383	1390	β-Elemene	0.6 ± 0.1	-	RI, MS
1414	1419	β-Caryophyllene	-	0.3 ± 0.0	RI, MS
1427	1434	α-*trans*-Bergamotene	0.2 ± 0.1	-	RI, MS
1450	1456	(E)-β-Farnesene	1.0 ± 0.1	-	RI, MS
1479	1480	ar-Curcumene	10.7 ± 0.2	1.4 ± 0.1	RI, MS
1492	1493	α-Zingiberene	24.9 ± 0.8	2.6 ± 0.1	RI, MS
1502	1505	β-Bisabolene	10.5 ± 0.3	0.6 ± 0.0	RI, MS
1523	1522	β-Sesquiphelladrene	11.9 ± 0.3	2.2 ± 0.0	RI, MS
Oxygenated sesquiterpenes			1.0 ± 0.2	73.9 ± 1.4	
1576	1583	ar-Turmerol	-	0.9 ± 0.0	RI, MS
1629	1628	1-*epi*-Cubenol	0.9 ± 0.2	-	RI, MS
1649	1646	Cubenol	0.2 ± 0.0	-	RI, MS
1677	1669	ar-Turmerone	-	38.7 ± 0.8	RI, MS
1681	-	α-Turmerone	-	14.2 ± 0.9	MS
1709	-	β-Turmerone	-	18.6 ± 0.6	MS
1742	1742	Bisabolone	-	0.7 ± 0.0	RI, MS
1778	1778	E-α-Atlantone	-	0.7 ± 0.0	RI, MS
Others			2.4 ± 0.1	-	
984	984	6-Methyl-5-Hepten-2-one	2.1 ± 0.1	-	RI, MS
1087	1087	2-Nonanone	0.1 ± 0.0	-	RI, MS
1287	1287	2-Undecanone	0.2 ± 0.0	-	RI, MS
		Total	94.6 ± 2.0	87.7 ± 0.7	

RI_Cal_: retention index relative to C_8_-C_32_ *n*-alkane on HP-5MSi column; RI_Ref:_ retention index reported in Adams, 2007; values are means ± standard deviation of three samples. Identification based on retention index (RI) and Mass spectra (MS) reported in NIST 11, Wiley 7n and literature.

**Table 2:** *In vitro* inhibitory effect of ginger and turmeric essential oils against *Portulaca oleracea*, *Lolium multiflorum*, *Echinochloa crus-galli*, *Cortaderia selloana* and *Nicotiana glauca* seed germination.

Seed Germination (% ± S.E.)					
Dose *	**Ginger essential oil**				
	***P. oleracea***	***L. multiflorum***	***E. crus-galli***	***C. selloana***	***N. glauca***
Control	86.00 ± 2.92 a	60.00 ± 2.74 a	86.00 ± 6.00 a	82.00 ± 3.74 a	94.00 ± 4.00 a
0.125	81.00 ± 4.30 a	50.00 ± 2.74 a,b	79.00 ± 3.67 a	85.00 ± 2.74 a	85.00 ± 5.48 a
0.25	77.00 ± 5.15 a	47.00 ± 5.61 a,b	73.00 ± 4.90 a	81.00 ± 3.32 a	83.00 ± 6.63 a
0.5	82.00 ± 2.55 a	47.00 ± 4.64 a,b	69.00 ± 5.79 a	67.00 ± 6.04 a	79.00 ± 11.34 a
1	47.00 ± 2.55 b	32.00 ± 8.89 b	68.00 ± 6.63 a	46.00 ± 6.21 b	73.00 ± 2.55 a
**Dose**	**Turmeric essential oil**				
Control	86.00 ± 2.92 a	60.00 ± 2.74 a	75.00 ± 7.01 a	82.00 ± 3.74 a	94.00 ± 4.00 a
0.125	75.00 ± 5.00 a	50.00 ± 3.87 a	74.00 ± 3.67 a	46.00 ± 15.12 a,b	85.00 ± 6.52 a
0.25	71.00 ± 2.45 a	49.00 ± 4.30 a	71.00 ± 2.92 a	43.00 ± 10.68 b	86.00 ± 2.92 a
0.5	70.00 ± 5.24 a	55.00 ± 3.54 a	71.00 ± 1.87 a	32.00 ± 6.82 b	87.00 ± 2.55 a
1	73.00 ± 4.06 a	49.00 ± 6.40 a	68.00 ± 2.55 a	15.00 ± 2.24 b	85.00 ± 2.24 a

Values are mean percentage of five replications ± standard error after 14 days of incubation. Means followed by different letters in the same column indicate that are significantly different at *p* < 0.05 according to T3 Dunnet and Tukey tests. * Dose: µL/mL.

**Table 3:** *In vitro* effects of ginger (G) and turmeric (T) essential oils on seedling length (hypocotyl and radicle) of *P. oleracea* (PO), *L. multiflorum* (LM), *E. crus-galli* (ECG), *C. selloana* (CS) and *N. glauca* (NG).

*Dose			Control	0.125	0.25	0.5	1
**G**	**PO**	Hyp	3.65 ± 0.22 a	2.80 ± 0.28 b	2.01 ± 0.12 c	1.39 ± 0.16 c,d	0.63 ± 0.09 d
		Rad	2.08 ± 0.26 a	2.07 ± 0.11 a	1.57 ± 0.21 a	0.89 ± 0.13 b	0.29 ± 0.09 b
	**LM**	Hyp	25.76 ± 0.90 a	19.65 ± 1.52 a,b	16.39 ± 3.58 b,c	12.46 ± 2.79 b,c	8.54 ± 3.16 c
		Rad	16.82 ± 1.93 a	10.67 ± 1.51 a,b	10.13 ± 2.12 a,b	6.69 ± 1.33 b	4.65 ± 1.85 b
	**ECG**	Hyp	16.96 ± 1.22 a	12.91 ± 0.33 a	12.88 ± 0.97 a	12.33 ± 1.82 a	12.27 ± 1.66 a
		Rad	13.24 ± 0.92 a	10.47 ± 0.89 a,b	9.01 ± 0.75 b	7.95 ± 1.30 b	6.54 ± 0.90 b
	**CS**	Hyp	4.14 ± 0.56 a	3.92 ± 0.70 a	2.74 ± 0.52 a,b	1.59 ± 0.71 b	1.09 ± 0.78 b
		Rad	3.88 ± 0.36 a	3.68 ± 0.50 a	2.63 ± 0.31 a,b	1.56 ± 0.21 b,c	0.96 ± 0.26 c
	**NG**	Hyp	4.72 ± 0.30 a	3.26 ± 0.40 a,b	2.99 ± 0.48 a,b	1.86 ± 0.57 b	1.71 ± 0.22 b
		Rad	3.87 ± 0.23 a	3.22 ± 0.24 a,b	3.37 ± 0.53 a,b	2.74 ± 0.70 a,b	2.00 ± 0.15 b
**T**	**PO**	Hyp	3.65 ± 0.22 a	1.97 ± 0.21 b	1.76 ± 0.13 b	1.51 ± 0.06 b	1.59 ± 0.04 b
		Rad	2.09 ± 0.26 a	2.32 ± 0.20 a	1.62 ± 0.18 a	1.53 ± 0.29 a	1.44 ± 0.12 a
	**LM**	Hyp	25.76 ± 0.90 a	15.34 ± 2.96 b	16.99 ± 1.41 b	16.85 ± 1.01 b	17.20 ± 1.62 b
		Rad	16.82 ± 1.93 a	11.60 ± 1.62 b	10.31 ± 1.14 b	10.70 ± 1.10 b	10.640.64 b
	**ECG**	Hyp	16.96 ± 1.22 a	11.35 ± 1.42 b	11.19 ± 1.01 b	10.37 ± 0.58 b	10.29 ± 0.86 b
		Rad	13.24 ± 0.92 a	9.80 ± 0.97 b	9.62 ± 0.60 b	8.27 ± 0.50 b	7.36 ± 0.82 b
	**CS**	Hyp	4.14 ± 0.56 a	1.57 ± 0.65 b	1.12 ± 0.47 b	0.69 ± 0.23 b	0.09 ± 0.05 b
		Rad	3.88 ± 0.36 a	0.88 ± 0.48 b	0.72 ± 0.29 b	0.54 ± 0.16 b	0.01 ± 0.01 b
	**NG**	Hyp	4.72 ± 0.30 a	1.82 ± 0.48 b	1.31 ± 0.24 b	1.15 ± 0.16 b	0.65 ± 0.17 b
		Rad	3.87 ± 0.23 a	2.55 ± 0.34 b	2.86 ± 0.09 b,c	2.40 ± 0.16 b,c	1.88 ± 0.12 c

Values are mean of five replications ± standard error after 14 days of incubation. Means followed by different letters in the same row indicate that are significantly different at *p* < 0.05 according to T3 Dunnet and Tukey tests. *Dose: µL/mL; Hyp: Hypocotyl (mm); Rad: Radicle (mm).

**Table 4:** *In vitro* seed germination and hypocotyl and radicle growth of tomato (To) cucumber (C) and rice (R) with ginger (G) and turmeric (T) essential oils.

* Dose			Control	0.125	0.25	0.5	1
**G**	**To**	Ger	70.00 ± 5.48 a	69.00 ± 6.60 a	66.00 ± 7.97 a	56.00 ± 5.79 a	54.00 ± 3.32 a
		Hyp	12.13 ± 0.80 a	8.76 ± 1.19 a,b	7.60 ± 1.37 b	3.32 ± 0.40 c	2.85 ± 0.57 c
		Rad	13.64 ± 1.41 a	10.88 ± 1.04 a,b	8.67 ± 1.56 b,c	6.12 ± 0.94 c,d	3.41 ± 0.37 d
	**C**	Ger	98.00 ± 1.23 a	95.00 ± 2.74 a	97.00 ± 2.00 a	96.00 ± 2.45 a	91.00 ± 2.45 a
		Hyp	10.34 ± 0.33 a	10.48 ± 0.17 a	10.10 ± 0.52 a	11.23 ± 0.78 a	11.75 ± 1.09 a
		Rad	18.61 ± 0.29 a	16.16 ± 0.54 a,b	16.57 ± 0.85 a,b	14.77 ± 0.74 b	14.62 ± 1.19 b
	**R**	Ger	97.00 ± 2.00 a	91.00 ± 1.87 a	94.00 ± 2.45 a	92.00 ± 1.23 a	91.00 ± 1.87 a
		Hyp	19.75 ± 2.58 a	21.78 ± 1.99 a	25.07 ± 1.31 a	20.05 ± 1.05 a	19.01 ± 1.02 a
Dose			Control	0.125	0.25	0.5	1
**T**	**To**	Ger	93.00 ± 1.23 a	85.00 ± 5.24 a	85.00 ± 5.24 a	78.00 ± 5.39 a	78.00 ± 5.15 a
		Hyp	12.64 ± 1.58 a	9.91 ± 1.92 a	8.62 ± 0.58 a	7.03 ± 0.93 a	8.77 ± 1.61 a
		Rad	18.13 ± 1.01 a	14.52 ± 1.81 a	14.35 ± 0.26 a	15.66 ± 3.23 a	10.11 ± 1.77 a
	**C**	Ger	98.00 ± 1.23 a	92.00 ± 2.55 a	96.00 ± 1.87 a	100.00 ± 0.00 a	97.00 ± 2.00 a
		Hyp	10.34 ± 0.33 a	10.38 ± 0.55 a	10.42 ± 0.71 a	9.57 ± 0.76 a	9.67 ± 0.08 a
		Rad	18.61 ± 0.29 a	17.61 ± 0.94 a	17.67 ± 0.28 a	17.00 ± 0.83 a	16.12 ± 0.51 a
	**R**	Ger	97.00 ± 2.00 a	92.00 ± 1.23 a	94.00 ± 2.92 a	94.00 ± 2.45 a	96.00 ± 1.87 a
		Hyp	19.75 ± 2.58 a	25.18 ± 1.12 a	26.83 ± 1.64 a	22.15 ± 1.92 a	21.19 ± 2.06 a

Values are mean of five replications ± standard error after 14 days of incubation. Means followed by different letters in the same row indicate that are significantly different at *p* < 0.05 according to T3 Dunnet and Tukey tests. *Dose: µL/mL; Hyp: Hypocotyl (mm); Rad: Radicle (mm).
